# Effects of nonpharmacological interventions on symptom clusters in breast cancer survivors: A systematic review of randomized controlled trials

**DOI:** 10.1016/j.apjon.2024.100380

**Published:** 2024-01-11

**Authors:** Meng-Yuan Li, Li-Qun Yao, Xian-Liang Liu, Jing-Yu (Benjamin) Tan, Tao Wang

**Affiliations:** a School of Nursing, Faculty of Health, Charles Darwin University, Brisbane, QLD, Australia; b School of Nursing and Health Studies, Hong Kong Metropolitan University, Hong Kong SAR, China; c School of Nursing and Midwifery, University of Southern Queensland, Ipswich, QLD, Australia

**Keywords:** Breast neoplasms, Symptom cluster, Nonpharmacological interventions, Systematic review

## Abstract

**Objective:**

To summarize nonpharmacological interventions and assess their effects on symptom clusters and quality of life (QoL) in breast cancer (BC) survivors.

**Methods:**

Seven English and three Chinese electronic databases and three clinical trial registries were searched from January 2001 to August 2023. A narrative approach was applied to summarize the data. The primary outcome was symptom clusters measured by any patient-reported questionnaires, and the secondary outcomes were QoL and intervention-related adverse events.

**Results:**

Six published articles, one thesis, and one ongoing trial involving 625 BC survivors were included. The fatigue-sleep disturbance-depression symptom cluster was the most frequently reported symptom cluster among BC survivors. The nonpharmacological interventions were potentially positive on symptom clusters and QoL among the BC survivors. However, some of the included studies exhibited methodological concerns (e.g., inadequate blinding and allocation concealment). The intervention protocols in only two studies were developed following a solid evidence-based approach. Adverse events related to the targeted interventions were reported in six included studies, with none performing a causality analysis.

**Conclusions:**

The nonpharmacological interventions could be promising strategies for alleviating symptom clusters in BC survivors. Future studies should adopt rigorously designed, randomized controlled trials to generate robust evidence.

**Systematic review registration:**

INPLASY202380028

## Introduction

Breast cancer (BC) has now surpassed lung cancer to be the most diagnosed cancer, accounting for one in eight cancer diagnoses globally.^1^ As per the report by the World Health Organization (WHO) in 2023, there were estimated 2.3 million cases of BC that occur annually,^2^ and this figure will increase by over 40% in 2040, reaching three million new cases annually.^1^ With the advances in public awareness, early screening, and improved treatments, the 5-year survival rate for BC survivors has significantly increased, ranging from 75% to 99% for those diagnosed at stages I to III.^3^ At the end of 2020, a globally estimated 7.8 million women have been living with a history of BC.^1^^,^^4^ Despite the fact that patients diagnosed with BC may have an improved survival rate, survivorship still experiences multiple psychosocial and physical symptoms, such as sleep distress, pain, depression, and fatigue, resulting from disease trajectories and/or cancer-related treatments.^5^ In studies of BC survivors, the pooled prevalence rates of these symptoms are as follows: 21.8% for persistent pain after treatment,^6^ 40% for sleep disturbance,^7^ 26.9% for severe fatigue,^8^ and 32.2% for depression.^9^ These symptoms are a significant source of distress and produce substantially negative health impacts, including reduced quality of life (QoL), impaired functional status, and low compliance with cancer treatment.^10^^,^^11^

Most of the time, these symptoms are identified and managed individually, despite the fact that they seldom occur in isolation but rather as a cluster. A symptom cluster was first defined as the co-occurrence of three or more associated symptoms by Dodd et al. in 2001.^12^ Several studies have revealed that the fatigue-sleep disturbance-depression (FSD) symptom cluster is highly prevalent, occurring in over 80% of BC survivors,^13^^,^^14^ severely affecting the survivor's life and mental status.^15^ Moreover, the symptom cluster of fatigue, pain, and psychological distress may also represent a substantial symptom burden, leading to a detrimental effect on both QoL and functional performance of BC survivors.^16^ Due to the dynamic constructs of the symptoms, the composition of a symptom cluster tends to change across different stages of the treatment trajectory. In some studies, clusters of psychoneurological and gastrointestinal symptom also have been reported.^17^^,^^18^ A symptom cluster may have shared natural associations and underlying mechanisms.^19^ When symptoms “cluster,” they will intensify one another through key events of cancer treatment types, tumor biology, and individual behavioral, psychological, or sociocultural factors.^20^ This collective impact of a symptom cluster can produce a greater negative cumulative effect than each of the individual symptoms on the daily life and functioning in BC survivors.^21^

Currently, targeted medications mainly have been recommended for managing individual cancer-related symptoms, such as the National Comprehensive Cancer Network (NCCN) Clinical Practice Guidelines in Oncology, which focus on addressing fatigue and pain separately among cancer patients.^22^^,^^23^ Nevertheless, recommendations for pharmacological interventions to manage the entire symptom cluster have been lacking. Meanwhile, some pharmacological agents for controlling individual symptoms may inadvertently exacerbate other symptoms or produce new symptoms. For example, using opioids to control pain may come with nausea and constipation.^24^ Moreover, the potential drug interactions with antineoplastic drugs also should not be ignored.^10^ Furthermore, the high cost of certain symptom control medications could contribute to economic hardship for cancer survivors.^25^ It is therefore important to understand what types of interventions are likely to be effective not only in addressing symptom clusters, but in considering cost and safety. For these reasons, nonpharmacological interventions have gained increasing attention as effective strategies to manage cancer-related symptoms. As an adjuvant to conventional pharmacological therapies, a nonpharmacological intervention “does not involve taking medicines or any other active agents” ^26^. Specifically, nonpharmacological interventions are nonmedicinal measures that are used in clinical practice and cover a wide range of interventions such as physical exercises, mindful-based interventions, Traditional Chinese Medicine (TCM), education-based interventions, etc. An increasing number of randomized controlled trials (RCTs) have found that nonpharmacological interventions could improve cancer survivors' symptom management, reduce the use of medications, and have fewer side effects.^27^^,^^28^

To date, two systematic reviews published in 2023^29^ and 2022^30^ identified preliminary evidence on using nonpharmacological interventions in the management of cancer-related symptom clusters; however, both only focused on interventions targeting a specific symptom cluster (e.g., FSD symptom cluster), and the value of nonpharmacological interventions on patients’ QoL was not explored in one of the studies.^29^ Most importantly, those two systematic reviews seem to have included studies based on predefined symptom clusters. Yet upon rigorous examination, it is apparent that these so-called clusters are typically individual symptoms rather than authentic symptom clusters.^29^^,^^30^ This systematic review was therefore conducted to: (1) follow the definition of symptom cluster to include eligible studies; (2) examine and assess the methodological quality of the included studies; (3) evaluate the effectiveness and safety of interventions that examined the impact on symptom clusters and QoL among BC survivors; and (4) identify the gaps that exist in the literature in order to provide implications for future research.

## Methods

The protocol of this review has been registered on the INPLASY platform (INPLASY202380028).^31^ This systematic review was performed according to the Joanna Briggs Institute (JBI) methodology and PRISMA reporting guidelines (Supplementary File 1).^32^

### Eligibility criteria

The PICOS tool (Participants, Intervention, Comparison, Outcomes, and Study Design) format was used to develop and present the eligibility criteria.

### Participants

Study participants were diagnosed with BC (≥ 18 years old), regardless of the stage of cancer (stages 0 to IV) or types of treatment (e.g., surgery, hormonal therapy, chemotherapy, or radiotherapy). Participants have suffered or reported symptom clusters, characterized by a clear definition of the co-occurrence of three or more symptoms,^12^ such as the FSD symptom cluster^30^ and the pain-fatigue-psychological distress symptom cluster.^33^

### Interventions

The interventions must be nonpharmacological interventions. Nonpharmacological interventions in this review were defined as any interventions that did not involve any medicines or any other active agents (e.g., consulting, acupressure, yoga, Qigong, relax therapies),^26^ aiming to manage symptom clusters either independently or along with routine methods of care.^34^

### Comparator

Comparisons involved either of the following: routine methods of care and/or standard medication, other nonpharmacological interventions, and/or pharmacological interventions or no intervention, or waitlist control.

### Types of studies

The types of studies were limited to RCTs with full text. The types of publications were in English peer-reviewed journals and core Chinese journals identified by the Institute of Scientific and Technical Information of China.

### Outcomes

The primary outcome of this study was the symptom cluster, referring to the term “cluster” or its synonyms. Symptoms within a cluster can be measured individually using any symptom-specific measures (e.g., the Multidimensional Fatigue Inventory for fatigue) or collectively based on their presence and severity (e.g., the Numerical Rating Scales, NRS). The QoL and safety outcomes, including any nonpharmacological interventions-related adverse events, were the secondary outcomes.

### Search strategy

Ten electronic databases, including the Chinese Biomedical Literature Database, Wan Fang Data, China National Knowledge Infrastructure (CNKI), PubMed, Cochrane Library, Web of Science, PsycINFO, Ovid Medline, Cumulative Index to Nursing and Allied Health Literature (CINAHL), and Excerpta Medica database (EMBase), were systematically searched for potentially eligible RCTs. Mesh terms, keywords, and free words such as “breast neoplas∗,” “symptom cluster∗,” “coexisting symptoms,” “randomized controlled trial,” “random∗,” “control∗” were used in the search strategies. Given that the concept of “symptom clusters” was initially introduced in 2001^12^, literature searching was therefore limited from January 2001 to August 2023. In addition, gray literature, including unpublished dissertations and conference summaries, was searched through the authors’ university library. The WHO International Clinical Trial Registry, ClinicalTrials.gov, and metaRegister of Controlled Trials (mRCT) were searched for ongoing trials. Additional records were identified by reviewing reference lists of the included studies. Details of the search strategies across databases were reviewed by two authors (Supplementary File 2).

### Study selection and data extraction

Data screening were conducted using EndNote 20.0 software, and duplicate records, were removed. Then, two authors (MYL and LQY) independently screened the titles and abstracts of the remaining records. After that, the same two authors (MYL and LQY) assessed the potential full text and determined its eligibility. Data extraction of each included study was performed by two authors (MYL and LQY) separately. The following data were extracted by a predefined data extraction form: (1) basic study details (publication year, first author, region), characteristics of participants (sample size, cancer stages, etc.); (2) details of nonpharmacological intervention protocols (instruction trainer, practitioner, intervention formula, frequency, and duration) and the control groups; (3) details of methodological quality (e.g., randomization and blinding); (4) study outcomes (primary and secondary outcomes associated with this systematic review). Any disagreements during the data screening and extraction were addressed through discussion between the two authors (MYL and LQY) to reach a consensus. If disagreement could not be addressed, the third author (TW) was involved in the final decision.

### Quality appraisal of the included studies

The revised JBI critical appraisal tool for RCTs was used to assess the methodological quality and risk of bias of each included study.^35^ The assessment tool consists of 13 questions:^35^ (1) “Was true randomization used for assignment of participants to treatment groups?”; (2) “Was allocation to treatment groups concealed?”; (3) “Were treatment groups similar at the baseline?”; (4) “Were participants blind to treatment assignment?”; (5) “Were those delivering treatment blind to treatment assignment?”; (6) “Were treatment groups treated identically other than the intervention of interest?”; (7) “Were outcome assessors blind to treatment assignment?”; (8) “Were outcomes measured in the same way for treatment groups?”; (9) “Were outcomes measured in a reliable way?”; (10) “Was follow-up complete, and if not, were differences between groups in terms of their follow-up adequately described and analyzed?”; (11) “Were participants analyzed in the groups to which they were randomized?”; (12) “Was appropriate statistical analysis used?”; (13)“ Was the trial design appropriate and any deviations from the standard RCT design (individual randomization, parallel groups) accounted for in the conduct and analysis of the trial?”.^35^ Each question has four potential responses: “Yes,” “No,” “Unclear,” and “N/A.”^35^ Two authors (MYL and LQY) independently assessed the potential risk of bias in the included RCTs, with the exclusion of their own articles from the evaluation. Instead, their assessments were cross-evaluated by the third author (TW) and one of the two authors mentioned above. In the case of discrepancies or nonconsensus between the two authors, another author (JYT) was available for additional assessment. The risk of bias for a study was categorized based on the percentage of “Yes” (> 70% low; 50% to 69% moderate; < 50% high).^36^

### Data analysis

A narrative and descriptive analysis, instead of meta-analysis, was used in this review given the significant heterogeneous nature of the intervention types, intervention protocol, comparisons, and study outcomes. The extracted data were used to perform narrative subgroup analysis, with studies categorized based on intervention types and the reported symptom clusters. For each outcome (e.g., targeted symptom clusters, QoL, and safety outcome), the findings were descripted according to types of intervention and subsequently summarized by the reviewers (MYL and LQY).

## Results

### Selection of studies

**Fig. 1:**
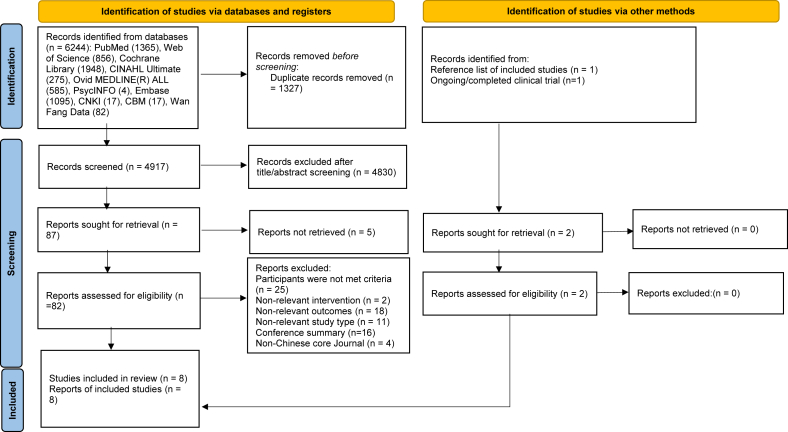
PRISMA flow diagram for study selection. *From:* Page MJ, McKenzie JE, Bossuyt PM, Boutron I, Hoffmann TC, Mulrow CD, et al. The PRISMA 2020 statement: an updated guideline for reporting systematic reviews. BMJ 2021;372:n71. doi: 10.1136/bmj.n71. For more information, visit: http://www.prisma-statement.org/. Notes: EMBase, Excerpta Medica database; CINAHL, Cumulative Index to Nursing and Allied Health Literature; CBM, Chinese Biomedical Literature Database; CNKI, China National Knowledge Infrastructure.

A total of 6246 relevant records were identified by initial searching the 10 databases (*n* = 6244) and other sources (manual retrieval, *n* = 2). After duplicates were removed and titles/abstracts were screened by End Note 20.0 software, 6157 records were removed. Eighty-nine potentially eligible records were reviewed in full text, of which 76 were excluded due to the following reasons: (1) participants were not meeting criteria (*n* = 25); (2) nonrelevant intervention (*n* = 2); (3) nonrelevant outcomes (*n* = 18); (4) nonrelevant study type (*n* = 11); (5) conference summary (*n* = 16); (6) no Chinese core journal (*n* = 4). Ultimately, six published articles, one thesis, and one registered ongoing trial were included (Fig. 1).

### Study characteristics

The six journal articles were published between 2016 and 2023,^37^, ^38^, ^39^, ^40^, ^41^, ^42^ the thesis was completed in 2022,^43^ and the registered ongoing trial was initially registered in 2021.^44^ Of the eight included studies, five were in English^38^, ^39^, ^40^, ^41^^,^^44^ and three were in Chinese.^37^^,^^42^^,^^43^ Studies were conducted in Mainland China (*n* = 6), the United States (*n* = 1), and Hong Kong SAR (*n* = 1). The eight included RCTs comprised 625 randomized BC survivors with different cancer stages, and 522 of them completed the studies (completed rate = 90.94%), except for the registered ongoing trial. The sample size of each RCT ranged from 31 to 180. Only two studies had more than 100 participants.^37^^,^^42^ One of the included RCTs comprised four arms,^42^ three RCTs comprised three arms,^37^^,^^38^^,^^44^ and the remaining RCTs comprised two arms.^39^, ^40^, ^41^^,^^43^

All included studies used a self-report instrument to assess BC survivors' outcomes and determine the intervention's effect. The individual symptoms most evaluated in the eight studies include fatigue (*n* = 8), sleep disturbance (*n* = 8), depression (*n* = 5), and pain (*n* = 4). The most commonly used instruments were the Brief Fatigue Inventory (BFI) for fatigue (*n* = 4), the Pittsburgh Sleep Quality Index (PSQI) for sleep disturbance (*n* = 6), the Hospital Anxiety and Depression Scale-Depression (HADS-D) for depression (*n* = 3), and the Brief Pain Inventory (BPI) for pain (*n* = 2). The effectiveness of the interventions on the FSD symptom cluster was specifically assessed in three studies.^38^, ^39^, ^40^ Other symptom clusters investigated in the remaining studies included the pain-fatigue-sleep disturbance (PFS) symptom cluster^41^^,^^44^ and the fatigue-pain-sleep disturbance-anxiety-depression (FPSAD) symptom cluster.^37^^,^^43^ Only one study hasn't analyzed a specific symptom cluster, but the commonly reported individual symptoms within the symptom clusters.^42^ In addition, four of the seven completed RCTs further utilized a correlation coefficient to confirm the presence of the examined symptom clusters.^38^, ^39^, ^40^, ^41^

Mostly, outcomes were assessed before and immediately after the intervention.^38^^,^^42^, ^43^, ^44^ Four studies conducted a follow-up assessment postintervention (ranging from one month to three months), evaluating the enduring effects of the nonpharmacological interventions.^37^^,^^39^, ^40^, ^41^ Additionally, two included studies conducted outcome assessments one or more times across the intervention to assess the temporal changes in the outcomes.^37^^,^^41^ Table 1 summarizes the characteristics of the included studies.

**Table 1:** Characteristics of included studies.

First author	Year	Region	Study design	Cancer stage	Sample size and age [year, (Mean ± SD)]	Intervention group (IG)	Control group (CG)	Outcomes measures (related to this systematic review topic only)	Symptom cluster measured	Data collection timing
A.X. Jin^37^	2021	Mainland China	Sham-controlled RCT∗	Stage 0 to III	**Randomized**: 180 **Completed**: 170 (analyzed) **Group A**: 58/60, age = 47.26 ± 6.24 **Group B**: 56/60, age = 46.30 ± 6.95 **Group C**: 56/60, age = 47.27 ± 5.96	**Group A**: Augmented reality exercise + manual acupressure on acupoints on the targeted symptom clusters.	**Group B**: Manual acupressure on acupoints on the targeted symptom clusters. **Group C**: Sham manual acupressure on acupoints nonrelevant to the targeted symptom clusters.	- **Fatigue**: BFI - **Pain**: BPI - **Sleep disturbance**: PSQI - **Depression**: SDS - **Anxiety**: SAS - **Safety**: Adverse events	**FPSAD symptom cluster**: Assessed using BFI, BPI, PSQI, SAS, and SDS concurrently.	At baseline, weeks 4, week 8, and week 12.
M.Y. Li^38^	2023	Mainland China	Partially blinded, sham controlled RCT∗	Stage I-IIIa	**Randomized**: 51; Completed: 45, 47 (analyzed) **Group A**: 16/17, age = 50.0 ± 6.75 **Group B**:15/17, age = 51.5 ± 9.5 **Group C**: 16/17, age = 53.5 ± 13.25	**Group A**: Manual acupressure with an education booklet on the management of cancer symptoms + usual care.	**Group B**: Sham manual acupressure only on non-acupoints away from meridians without evoking the “Deqi.” sensation + an education booklet on the management of cancer symptoms + usual care. **Group C**: Usual care + an education booklet on the management of cancer symptoms.	- **Fatigue**: MFI - **Sleep disturbance**: PSQI - **Depression**: HADS-D - **QoL**: FACT-B - **Safety**: Adverse events	- **FSD symptom cluster**: The MFI, HADS-D, and PSQI total scores were rescaled into 0–10 NRS (0 = ‘no’, 10 = ‘as bad as it could be’) respectively; then the average of the three symptoms on 0–10 NRS was taken as the entire symptom cluster severity. - Regression coefficients of GEE model were used to identify the association among the fatigue, depression and sleep disturbance.	At baseline, and postintervention (7 weeks).
W. M. Wong^39^	2023	Hong Kong SAR	Single-blind RCT∗	Stage I to III	**Randomized**: 50 (analyzed) **Completed**: 47 **IG**:24/25, age = 51.70 ± 10.5 **CG:**23/25, age = 56.60 ± 10.9	An oncology nurse-led intervention program, including psychological support and education on chemotherapy side effects, how to manage them, and dietary and exercise recommendations.	Usual care: 10-min face-to-face education and paper materials on managing chemotherapy-related side effects.	- **Fatigue**: BFI - **Sleep disturbance**: PSQI - **Depressed mood**: CES-D - **QoL**: FACT-B - **Safety**: Adverse events	- **FSD symptom cluster**: All three individual symptoms of FSD occur simultaneously. The cut-offs were a PSQI score ≥ 5, a BFI score ≥ 1, and a CES-D score ≥ 16. - Correlation was identified between fatigue, depressed mood and sleep disturbance.	At baseline postintervention (7 weeks) and 3 months following the intervention.
L.Q. Yao^40^	2022	Mainland China	RCT∗	Stage I-IIIa	**Randomized**: 72 (analyzed) **Completed**: 69 **IG**:36/36, age = 45.3 ± 8.5 **CG**:33/36, age = 48.6 ± 7.8	Tai chi (8-form yang style) with an education booklet on the management of symptom cluster + routine care.	Routine care + an education booklet on the management of symptom cluster.	- **Fatigue**: BFI - **Depression**: HADS-D - **Sleep disturbance**: PSQI - **QoL**: FACT-B - **Safety**: Adverse events	- **FSD symptom cluster**: Using the PSQI, BFI and HADS-D concurrently. - The regression coefficients of an adjusted GEE model were used to identify the association among depression, fatigue, and sleep disturbance.	At baseline, postintervention (8 weeks), and 1-month follow-up.
C.H. Yeh^41^	2016	USA	RCT∗	Any stage	**Randomized**: 31 (analyzed), age = 58.32 ± 10.93 **Completed**: 25 **IG**: 14/16 **CG**: 11/15	- Auricular acupressure on the acupoints related to the targeted symptom cluster. - Plant seeds were placed on the designated acupoints using tape.	- Auricular acupressure on the acupoints unrelated to the targeted symptom cluster. - Plant seeds were placed on the designated acupoints using tape.	- **Individual symptoms**: MDASI - **QoL**: WHOQOL-BREF - **Safety**: Adverse events	- **PFS symptom cluster** was measured using MDASI. - The **pearson correlation coefficient** was used to identify the correlation among pain, sleep disturbance and fatigue.	At baseline, week 1, week 2, week 3, week 4 and 1-month follow-up.
Y.L. Zhang^42^	2016	Mainland China	RCT∗	NR	**Randomized**: 144, age = 49.0 ± 7.4 **Completed**: 128 (analyzed) **Group A**: 32/36 **Group B**: 30/36 **Group C**: 32/36 **Group D**: 34/36	**Group A**: Aerobic exercise + usual care **Group B**: Relaxation therapy + usual care **Group C**: Aerobic exercise + relaxation therapy + usual care	**Group D**: Usual care	**Individual symptoms**: MSAS **QoL**: FACT-B	**Symptom clusters**: Assessed using MSAS and reported the co-existing symptoms.	At baseline and postintervention (6 weeks).
Q.Q. Wang^43^	2022	Mainland China	RCT∗∗	Stage I to III	**Randomized**: 46 **Completed**: 38 (analyzed) **IG**: 19/23, age = 56.11 ± 3.56 **CG**: 19/23, age = 57.58 ± 3.83	Combined aerobic exercise and resistance exercise with elastic bands + routine care.	Routine care	- **Pain**: VAS - **Fatigue**: RCFS-CV - **Sleep disturbance**: PSQI - **Depression and anxiety**: HADS - **QoL**: FACT-B - **Safety**: Adverse events	**Pain-fatigue-sleep disturbance-anxiety-depression symptom cluster**: Assessed using VAS, RCFS-CV, PSQI, HADS concurrently.	At baseline and postintervention (8 weeks).
H.L. Cheng^44^	2021	Mainland China	RCT∗∗∗	Stage I to IIIa	**Randomized**: 51 **Group A**: 17 **Group B**: 17 **Group C**: 17	**Group A**: Manual self-acupressure on acupoints.	**Group B**: Manual sham acupressure on nonacupoints, **Group C**: Routine care	- **Arthralgia**: BPI - **Fatigue**: BFI - **Sleep disturbance**: PSQI - **QoL**: FACT-G	NA	At baseline and postintervention (6 weeks).

MDASI, M. D. Anderson Symptom Inventory; WHOQOL-BREF, World Health Organization Quality of Life; BFI, Brief Fatigue Inventory; PSQI, Pittsburgh Sleep Quality Index; HADS-D, Hospital Anxiety and Depression Scale-Depression; HADS, Hospital Anxiety and Depression Scale; FACT-B, the Functional Assessment of Cancer Therapy-Breast; MFI, Multidimensional Fatigue Inventory; CES-D, 20-item Center for Epidemiologic Studies–Depression; BPI, Brief Pain Inventory; SAS, Self-Rating Anxiety Scale; SDS, Self-Rating Depression Scale; MSAS, Memorial Symptom Assessment Scale; VAS, Visual Analogue Scale; RCFS-CV, Revised Piper Fatigue Scale-Chinese Ver; FACT-G, Functional Assessment of Cancer Therapy scale; SAR, Special administrative region; FSD, Fatigue-sleep disturbance-depression; PFS, pain-fatigue-sleep disturbance; FPSAD, fatigue-pain-sleep disturbance-anxiety-depression; NRS, Numerical Rating Scale; IG, Intervention group; CG, Control group; NR, Not reported; NA, Not applicable; ∗, Published study; ∗∗, Master thesis, ∗∗∗, Registered ongoing study.

### Intervention protocols

A full detail of the interventions utilized in the included studies is provided in Table 2, including intervention types, procedures, instruction trainers, and timing, duration, and frequency of the interventions. Considering the notable clinical heterogeneity observed in the reported interventions in the studies, they were categorized as follows: multimodal interventions (nurse-led multi-modal intervention program; augmented reality (AR)-assisted self-acupressure and exercise; the combination of aerobic exercise and relaxation therapy),^37^^,^^39^^,^^42^ mind-body exercise (Tai chi),^40^ physical-based exercise (aerobic and resistance exercises),^43^ and TCM (somatic acupressure, auricular acupressure).^38^^,^^41^^,^^44^ The most commonly reported intervention timing is during the chemotherapy. The registered ongoing trial involved BC survivors who had completed cancer-related treatments,^44^ while one study did not specify the timing of intervention.^37^ Of the seven studies that used self-practice intervention, the most commonly reported instruction trainers were either study investigators (*n* = 4) or qualified registered nurses (*n* = 2) or a combination of both (*n* = 1). The remaining study used a nurse-led multi-modal intervention program.^39^ The interventions of four studies were developed based on certain theories or frameworks. The development of an auricular point acupressure intervention by Yeh et al. was reflex theory-driven.^41^ Li et al.^38^ and Yao et al.^40^ applied the Medical Research Council (MRC) Framework for Developing and Evaluating Complex Interventions. Additionally, neurophysiological theories and some TCM theories (e.g., *yin-yang* theory, inflammatory theory, and *zang-fu* organs and meridians theory) were identified and utilized to clarify the potential mechanisms of the described interventions in those two studies in managing the targeted symptom clusters in BC survivors.^38^^,^^40^ The intervention protocols in those two studies were also evidence-based.^38^^,^^40^ Wong et al^39^ used the Predisposing, Reinforcing, and Enabling Constructs in Educational Diagnosis and Evaluation (PRECEDE)–PROCEED model for the development of interventions to improve symptoms. The conceptual framework of neuroendocrine-immune mechanisms and mediators of psychopathology was used by Wang^43^ to clarify the relationship between the targeted intervention and its outcomes.

**Table 2:** Intervention protocol of included studies.

Study	Type	Procedure	Instruction trainer	Self-practice (Y/N)	Timing	Duration	Frequency	Follow-up
A.X. Jin^37^	- AR-assisted exercise: Aerobic exercise and resistance exercise - AR-assisted self-acupressure	- The patient stands in the AR system's detection zone and starts the somatosensory exercise and acupressure software. - Start the AR exercise following the on-screen instructions. - After the exercise, start the self-manual acupressure on the selected acupoints following the on-screen instructions of the AR system. - The intensity of manual acupressure was evoking the soreness or numbness sensations.	Clinical nurse specialist.	Y	NR	8 weeks	- **AR exercise**: Aerobic exercise (5 days/week, once daily) a) Warm-up exercise (5 min/time) b) Endurance training (20–30 min/time) c) Cool-down exercise (5 min/time) Resistance training (2–3 times/week, 5–10 min/time) - **Manual acupressure**: 5 days/week, once daily within 30 min	1 month
M.Y. Li^38^	Manual acupressure	- Self-acupressure on the 11 selected acupoints with the fingers, evoking the sensation of “*Deqi*”. - Light self-acupressure at eleven nonacupoints, but without the sensation of “*Deqi*”.	Trained research assistant by a qualified acupuncture practitioner.	Y	After chemotherapy	7 weeks	7 times/week, a daily 36 min/time	N
W.M. Wong^39^	Nurse-led multimodal intervention program	- At week 1, a 25-min face-to-face education before chemotherapy, including the introduction and management of the FSD symptom cluster and chemotherapy side effects, as well as dietary and exercise recommendations. - At week weeks 2–3 and weeks 5–6, a 20-min telephone session 20-min telephone session focused on recommendations on diet and exercise, feedback and suggestions in managing the chemotherapy-related side effects. - At week 4 and 7, a routine assessment of the participants' FSD symptom cluster.	Nurses with a nursing master's degree and a minimum of five years of oncology experience.	N	Before or during chemotherapy	7 weeks	Weekly	3 months
L.Q. Yao^40^	Tai chi (8-form yang style)	- A 10-min warm-up. - Easy eight form Tai chi practising for 25–30 min, including commencing form (qishi), repulse monkey, (juangongshi), grasp peacock's tail (lanquewei), wave hands like clouds (yunshou), fair lady works at shuttles (zuoyouchuansuo), golden cock stands on one leg (jinjiduli), brush knees and twist steps (louxiaobu), closing form (shoushi). - A 10-min cool-down. - Rest for 10-min between each session.	Trained study investigator and clinical nurses by a qualified Tai chi instructor	Y	During chemotherapy	8 weeks	2 times/week, 60 min/time	1 month
C.H. Yeh^41^	Auricular point acupressure	- Plant seeds are attached with tape to specific acupoints to relieve pain, sleep disturbance and fatigue. - Press the seeds for moderate stimulation using the thumb and index finger.	Primary investigator assisting with an acupoint locator	Y	During or after chemotherapy/radiotherapy	4 weeks	5 days/week,3 times/day, 3 min/time	1 month
Y.L. Zhang^42^	Aerobic exercise + relaxation therapy	- **Aerobic exercise** a) provide a handbook on the benefits and techniques of aerobic exercise. b) Create a personalized exercise plan with a moderate intensity of 55%–65% of maximum heart rate. c) Choose the modalities according to individual preferences and conditions. - **Relaxation therapy** a) provide a leaflet on the process of relaxation therapy b) Explain the process of relaxation therapy c) Perform the self-relaxation following the provided self-relaxation tutorials (published by the Chinese Medical association Audio-Video press)	Study investigator	Y	During chemotherapy	6 weeks	- **Aerobic exercise**: 3–5 times/week, 20–30 min/time - **Relaxation therapy**: one time before sleep (length of one session was not reported)	N
Q.Q. Wang^43^	Aerobic exercise + resistance exercises with elastic bands	- Aerobic exercise: a 20–30 min walking - Resistance exercises with elastic bands a) a 5-min warm-up exercise b) Chest press (2 sets × 10–12 reps) c) Seated row (2 sets × 10–12 reps) d) Shoulder press (2 sets × 10–12 reps) e) Standing hip flexion (1 sets × 10–12 reps each side) f) Seated leg extension (2 sets × 10–12 reps) g) Standing hamstring curls (1 sets × 10–12 reps each side) h) A 5min cool-down	Study investigator	Y	During chemotherapy	8 weeks	- **Aerobic exercise**: 20–30 min/time (no more than 50 min), twice per week - **Resistance exercise with elastic bands**: Twice per week - Alternating between aerobic and resistance exercises weekly	N
H.L. Cheng∗^,^^44^	Manual acupressure	N	N	Y	After cancer-related treatment	6 weeks	N	N

AR, Augmented reality; Y, Yes; N, No; ∗, Registered ongoing trial.

### Methodological quality of included studies

Table 3 presents the risk of bias assessment of the included studies. Four studies^37^, ^38^, ^39^, ^40^ presented a low risk of bias, two studies presented a moderate risk,^41^^,^^43^ and one study presented a high risk.^42^ All seven studies reported proper randomization, including a computer-generated routine,^38^^,^^40^^,^^41^ a random sequence by an independent research assistant,^39^ and a random number table.^37^^,^^42^^,^^43^ Nevertheless, adequate concealment was described in only three studies using a sealed envelope or an independent random sequence keeper.^38^, ^39^, ^40^ For baseline characteristics among study groups, all studies showed comparable characteristics. One RCT described the partial blinding of outcome assessors and participants,^38^ while a double-blind design was difficult to achieve due to the visible nature of the intervention. Four of the studies used intention-to-treat analysis.^38^, ^39^, ^40^, ^41^ Only one study did not conduct the data analysis between groups postintervention.^42^

**Table 3:** Methodology quality of the included studies.^a^

Study	Internal Validity Bias										Statistical conclusion validity			Total of “Yes” scores (%)	Risk of bias
	Selection and allocation			Administration of intervention/exposure			Assessment, detection, and measurement of the outcome			Participant retention					
	Q1	Q2	Q3	Q4	Q5	Q6	Q7	Q8	Q9	Q10	Q11	Q12	Q13		
A.X. Jin^37^	Yes	Unclear	Yes	Unclear	No	Unclear	Unclear	Yes	Yes	Yes	No	Yes	Yes	7/13, 53.8%	Low
M.Y. Li^38^	Yes	Yes	Yes	Yes^b^	No	Yes	Yes^b^	Yes	Yes	Yes	Yes	Yes	Yes	12/13, 92.3%	Low
W.M. Wong^39^	Yes	Yes	Yes	No	No	Yes	No	Yes	Yes	Yes	Yes	Yes	Yes	10/13, 76.9%	Low
L.Q. Yao^40^	Yes	Yes	Yes	No	No	Yes	No	Yes	Yes	Yes	Yes	Yes	Yes	10/13, 76.9%	Low
C.H. Yeh^41^	Yes	Unclear	Yes	Unclear	No	Yes	Unclear	Yes	Yes	Yes	Yes	Yes	Yes	9/13, 69.2%	Moderate
Y.L. Zhang^42^	Yes	Unclear	Yes	No	No	Yes	No	Yes	Yes	Yes	No	No	Unclear	6/13, 46.1%	High
Q.Q. Wang^43^	Yes	Unclear	Yes	No	No	Yes	No	Yes	Yes	Yes	No	Yes	Yes	8/13, 61.5%	Moderate

“Q1. “Was true randomization used for assignment of participants to treatment groups?” Q2. “Was allocation to treatment groups concealed?” Q3. “Were treatment groups similar at the baseline?” Q4. “Were participants blind to treatment assignment?” Q5. “Were those delivering treatment blind to treatment assignment?” Q6. “Were treatment groups treated identically other than the intervention of interest?” Q7. “Were outcomes assessors blind to treatment assignment?” Q8. “Were outcomes measured in the same way for treatment groups?” Q9. “Were outcomes measured in a reliable way?” Q10. “Was follow up complete and if not, were differences between groups in terms of their follow up adequately described and analyzed?” Q11 “Were participants analyzed in the groups to which they were randomized?” Q12 “Was appropriate statistical analysis used?” Q13. “Was the trial design appropriate, and any deviations from the standard RCT design (individual randomization, parallel groups) accounted for in the conduct and analysis of the trial?” ——Source: Barker, T.H., Stone, J.C., Sears, K., Klugar, M., Tufanaru, C., Leonardi-Bee, J., Aromataris, E. and Munn, Z., (2023). The revised JBI critical appraisal tool for the assessment of risk of bias for randomized controlled trials. JBI Evidence Synthesis, 21(3): 494–506.

a The quality assessment was conducted on completed trials with the final results being published.

b Participants in the true and sham intervention groups were blinded. Since the outcome measures were patient-reported, blinding for the outcome's assessors for the true and sham intervention groups was also achieved.

### Effects of nonpharmacological interventions on symptom clusters and multiple symptoms

The effects of the nonpharmacological interventions on symptom clusters in BC survivors are summarized in Table 4. The effects of nonpharmacological interventions on the following three symptom clusters were evaluated in the seven included studies: the FSD symptom cluster,^38^, ^39^, ^40^ the PFS symptom cluster^41^^,^^44^ and the FPSAD symptom cluster.^37^^,^^43^

**Table 4:** Effects of nonpharmacological interventions on symptom cluster.

Study	Outcomes		Assessment time points	Description of the intervention's effects
A.X. Jin^37^	- BFI - BPI - PSQI - SAS - SDS	- Fatigue - Pain - Sleep disturbance - Anxiety - Depression	Week 4	- **Generalized linear mixed model**: Significant group-by-time interaction effect was found in the score of each symptom (fatigue, pain, sleep disturbances, anxiety, and depression) within the cluster (*P* < 0.001).
			Week 8	
			Week 12	
M.Y. Li^38^	- MFI - PSQI - HADS-D	- Fatigue - Sleep disturbance - Depression	Postintervention (7 weeks)	- The group-by-time effect on the composite score of the FSD symptom cluster was significant (*P* < 0.05).
W.M. Wong^39^	- BFI - PSQI - CES-D	FSD symptom cluster	Postintervention (7 weeks)	- The occurrence of the FSD symptom cluster was lower in the intervention group at postintervention (*P* = 0.480) and follow-up (*P* = 0.035) when compared with the control group and baseline - No statistically significant between-group difference in the change in the occurrence of the FSD symptom cluster postintervention. - The correlations between fatigue and sleep disturbance (*P* = 0.004), fatigue and depression (*P* = 0.001), and sleep disturbance and depression (*P* = 0.001) were moderately positive at baseline.
			3 months follow-up	
L.Q. Yao^40^	- BFI - PSQI - HADS-D	- Fatigue - Sleep disturbance - Depression	Postintervention (8 weeks)	- **Unadjusted GEE model**: The intervention group demonstrated statistically significant reductions in depression (*P* = 0.006), fatigue (*P* < 0.001), and sleep disturbance (*P* < 0.001) after intervention and follow-up compared with the control group and baseline. - **Unadjusted GEE model**: The clinically significant within-group differences were observed in the scores of BFI (1.93 and 3.24 points), PSQI (4.25 and 5.95 points), and HADS-D (3.47 and 4.58 points) from baseline to postintervention and from baseline to follow-up in the intervention group. - **Adjusted GEE model**: Sleep disturbance, fatigue, and depression showed significant correlation with each other (all at *P* < 0.05).
			4-week follow-up	
C.H. Yeh^41^	MDASI	- Pain - Fatigue - Sleep disturbance	Postintervention (8 weeks)	- Clinically significant reductions (defined as symptom decreases of ≥ 30%) of 31% in sleep disturbance, 44% in fatigue, and 71% in pain were observed in the intervention group from baseline to postintervention. - No statistically significant differences were identified between groups of sleep disturbance (*P* = 0.0642) and fatigue (*P* = 0.2351) postintervention, but significant improvement in pain (*P* = 0.0217) was observed postintervention. - Pain was highly associated with fatigue and sleep disturbance at baseline and postintervention (correlation coefficient ≥ 0.35). - No statistically significant differences were identified between groups of pain (*P* = 0.0767), fatigue (*P* = 0.1760), and sleep disturbance (*P* = 0.2875) after follow-up.
			1 month follow-up	
Y.L. Zhang^42^	MSAS	- Fatigue - Lack of appetite - Sleep disturbance - Taste change - Nausea	Postintervention (8 weeks)	- Significant improvement in the mean scores of fatigue and sleep disturbance in the group receiving aerobic exercise along with relaxation therapy postintervention relative to baseline (both *P* < 0.05). - Significant improvement in the mean scores of fatigue, sleep disturbance, and lack of appetite in the group only receiving aerobic exercise postintervention relative to baseline (all *P* < 0.05). - No statistically significant reduction of targeted symptoms in the group only receiving relaxation therapy over time (all *P* > 0.05), as well as in the control group (all *P* > 0.05).
Q.Q. Wang^43^	- VAS - RCFS-CV - PSQI - HADS-A - HADS-D	- Pain - Fatigue - Sleep disturbance - Anxiety - Depression	Postintervention (6 weeks)	- The intervention group showed more significant effects in reducing pain, fatigue, anxiety, and depression than the control group postintervention (all *P* < 0.05). - No significant between-group differences in the score of sleep disturbance (*P* > 0.05).

IG, Intervention group; CG, Control group; FSD symptom cluster, fatigue-sleep disturbance-depression symptom cluster; MDASI, M. D. Anderson Symptom Inventory; BFI, Brief Fatigue Inventory; PSQI, Pittsburgh Sleep Quality Index; HADS-A, Hospital Anxiety and Depression Scale-Anxiety; HADS-D, Hospital Anxiety and Depression Scale-Depression; HADS, Hospital Anxiety and Depression Scale, MFI, Multidimensional Fatigue Inventory; CES-D, 20-item Center for Epidemiologic Studies–Depression; BPI, Brief Pain Inventory; SAS, Self-Rating Anxiety Scale; SDS, Self-Rating Depression Scale; MSAS, Memorial Symptom Assessment Scale; VAS, Visual Analogue Scale; RCFS-CV, Revised Piper Fatigue Scale-Chinese Ver; GEE, generalized estimating equation.

#### The fatigue–sleep disturbance-depression symptom cluster

Three studies separately examined the effect of Tai Chi, somatic acupressure, and a nurse-led multi-modal intervention program on the FSD symptom cluster.^38^, ^39^, ^40^ All three studies reported a potentially positive statistical or clinical effect. Yao et al.^40^ reported clinically significant improvements in all three symptoms in the FSD symptom cluster among BC survivors who received eight weeks of Tai chi intervention over time. The unadjusted Generalized Estimating Equation (GEE) model also revealed statistically significant reductions in fatigue (*P* < 0.001), depression (*P* = 0.006), and sleep disturbance (*P* < 0.001) after intervention and follow-up compared with the control group and baseline. Similarly, Wong et al.^39^ reported positive findings on a multimodal intervention program. The occurrence of the FSD symptom cluster was lower in the intervention group at postintervention (*P* = 0.480) and follow-up (*P* = 0.035) when compared with the control group and baseline.^39^ Nevertheless, the between-group difference in change in log odds of the FSD symptom cluster at postintervention was not significant statistically.^39^ For the somatic acupressure outlined in one study, the group-by-time effect on the composite score of the FSD symptom cluster was statistically significant (*P* < 0.05); however, there were no significant differences in the FSD symptom cluster between groups at postintervention.^38^ Despite the variations in the findings of the three studies regarding the between-group comparisons, there was a positive trend indicating improvement in the FSD symptom cluster among those who received the interventions.

#### The fatigue-pain-sleep disturbance-anxiety-depression symptom cluster

Two of the seven completed trials^37^^,^^43^ investigated the effect of physical-based intervention (aerobic and resistance exercises) and multimodal interventions (aerobic exercise, resistance exercise, and acupressure assisting with augmented reality) on the FPSAD symptom cluster separately. An augmented reality-assisted exercise (aerobic and resistance exercises) and acupressure program was conducted by Jin et al.^37^ and led to the group-by-time interaction effect in the score of each symptom within the FPSAD symptom cluster (*P* < 0.001). Likewise, when Wang et al.^43^ evaluated the effectiveness of the intervention involving aerobic and resistance exercises in relieving the severity of the FPSAD symptom cluster, they found a significant between-group difference in pain, fatigue, anxiety, and depression (all *P* < 0.05) postintervention but not sleep disturbance (*P* > 0.05). Thus, fairly consistent results were obtained for the effects of the interventions on the FPSAD symptom cluster.

#### The pain-fatigue-sleep disturbance symptom cluster

Only one study assessed the effect of nonpharmacological interventions on the PFS symptom cluster.^41^ The M. D. Anderson Symptom Inventory (MDAS) was used to evaluate the severity and/or level of interference of the symptom cluster in patients’ daily lives. In this study focused on acupressure, a statistically significant difference was observed only in the alleviation of pain (*P* = 0.0217) but not fatigue (*P* = 0.2351) or sleep disturbance (*P* = 0.0642) when comparing the intervention group to the control group after the intervention. Yeh et al.^41^ also reported clinically significant reductions (defined as a reduction of 30% or more) in the three individual symptoms within the PFS symptom cluster in the intervention group postintervention. Overall, there was a reduction in the severity of pain within the PFS symptom cluster among individuals receiving the acupressure, suggesting its effectiveness in alleviating the symptom burden experienced among BC survivors.

#### Multiple symptoms from identified symptom clusters

The last remaining study^42^ examined the impact of combining aerobic exercise with relaxation therapy on the five most prevalent symptoms from the four identified symptom clusters. The assessment tool was the Memorial Symptom Assessment Scale (MSAS). This RCT found a significant decrease in sleep disturbance and fatigue among the intervention group (aerobic exercise combined with relaxation therapy) over time (both *P* < 0.05). Similarly, the severity of fatigue, lack of appetite, and sleep disturbance were significantly reduced (all *P* < 0.05) in those who only received aerobic exercise after intervention. However, there was no significant reduction in investigated symptoms in the group only receiving relaxation therapy over time (all *P* > 0.05), as well as in the control group (all *P* > 0.05).

### Effects of nonpharmacological interventions on quality of life

Six of the completed trials reported the effect of five different types of intervention on QoL. The assessment of QoL utilized the FACT-B^38^, ^39^, ^40^^,^^42^^,^^43^ and the WHO Quality of Life (WHOQOL-BREF).^41^ Yeh et al.^41^ reported that the auricular acupressure intervention yielded better QoL compared to the control group; however, the difference in the improvement did not reach statistical significance. In another study utilizing the somatic acupressure,^38^ the within-subject effects of time on QoL were significant (*P* < 0.001). In addition, the combination of aerobic exercise and relaxation therapy, as described by Zhang and Zhang (2016), demonstrated significant improvement in QoL within-group (*P* < 0.05), particularly in the domains of social (*P* < 0.05) and functional well-being (*P* < 0.05). Yao et al.^40^ observed that the QoL total score as measured by FACT-B in the Tai chi intervention group was significantly improved at postintervention (*P* = 0.032) and four weeks' follow-up (*P* < 0.001) than the control group and baseline. Generally, results from these studies indicated the potential beneficial effects of the five different nonpharmacological interventions on BC survivors’ QoL.

### Adverse events

Safety or the adverse event related to the intervention was monitored in six trials.^37^, ^38^, ^39^, ^40^, ^41^^,^^43^ Of which three studies involving the intervention of acupressure, aerobic exercise combined with resistance exercises, and a multi-modal intervention program reported no adverse effects,^38^^,^^39^^,^^43^ while one mentioned the monitoring of intervention safety during the intervention (AR-assisted exercise and self-acupressure) but did not report any details in the results section.^37^ Yeh et al.^41^ reported minimal adverse local effects from auricular acupressure weekly, such as ear pain, tenderness, discomfort, and itchiness following seed placement. However, these sensations gradually decreased throughout the intervention, and no participants were recorded as dropping out due to these adverse effects. Another study reported minor discomforts, and eight participants experienced minor knee or musculoskeletal pain during or after practicing Tai chi, but these uncomfortable reactions disappeared shortly after they stopped practicing or had a rest.^40^ None of the included studies examined causality between the reported adverse events and the utilized interventions.

## Discussion

### Main findings

Given the limited quantity of the included RCTs in this review and the moderate to high risk of bias in the three included RCTs, the current evidence may not be entirely conclusive but does indicate a beneficial role of nonpharmacological interventions in managing the symptom clusters in BC survivors. So et al.^28^ stated that the effectiveness of nonpharmacological interventions on cancer-related symptom clusters was related to the study participants, intervention characteristics, and outcome measures. The findings of this systematic review should, therefore, be prudent to interpret cautiously.

Meta-analysis has been deemed inappropriate for systematic reviews with substantial clinical heterogeneity in the included studies.^45^ The findings via descriptive analysis demonstrated an encouraging effect of nonpharmacological interventions on symptom management and overall QoL compared with usual care, sham control, etc. The FSD symptom cluster was the most frequently reported and generally observed downtrend in the reported studies.^38^, ^39^, ^40^ In accordance with the systematic reviews conducted by So et al.^28^ and He et al.^29^ this review suggested that the nonpharmacological interventions were potentially beneficial to the symptom clusters in BC survivors, including the FSD symptom cluster, the FPSAD symptom cluster, and the PFS symptom cluster. However, only one study assessed continuous effects with a follow-up period of three months after the intervention,^39^ indicating that solid evidence for the possible longer-term effects of nonpharmacological interventions remains inconclusive. The design of follow-ups after the intervention, particularly long-term follow-up (e.g., at least three months) assessments, is therefore recommended for future clinical trials to evaluate the continuous effects of the nonpharmacological interventions, which could further provide valuable evidence regarding the optimal treatment period of the targeted intervention to achieve the maximum beneficial effect. Also, findings from this review indicated that nonpharmacological interventions were generally safe adjunct treatments with no or very mild adverse events, which is consistent with a previous systematic review finding.^46^ Its relatively low-risk nature and minimal side effects could make it easier to be accepted by both clinical practitioners and cancer survivors.

Out of the seven trials completed, the interventions of four studies were theory-driven. Among these, two studies^38^^,^^40^ demonstrated the development and validation of an evidence-based intervention protocol for managing symptom clusters in BC survivors systematically following the MRC framework in the study of complex interventions. Developing an intervention protocol for managing symptom clusters in BC survivors, grounded in current theories and best evidence, is a distinguished feature to ensure that the study procedures are practically appropriate. Of the nonpharmacological intervention modalities, exercise-based interventions (aerobic and resistance exercises, Tai chi) and acupressure (e.g., auricular acupressure, somatic acupressure) were the most prevalent among the included trials. Nevertheless, determining the most effective and appropriate types of nonpharmacological interventions for the same symptom cluster, restricted to a small number of studies, was not feasible. Additionally, variation in the duration and frequency of the same or similar types of interventions was observed, suggesting the absence of standardized practice with evidence-based components. Based on the descriptive analysis, it appeared feasible to perform somatic acupressure and exercise-based interventions for six to eight weeks, however, no consistent interventions related to somatic acupressure or exercises have been observed with sufficient sample sizes. The heterogeneity of the participants may also contribute to discrepancies in the intervention effects reported. Participants diagnosed with BC received different treatment types, were at varying stages of the disease trajectory, and had different symptom experiences and severity. These findings could be attributed to the variations in interventions tailored to address different concurrent symptoms or symptom clusters under the different etiologies.

### Implications for clinical practice and research

Previous guidelines and studies have mainly focused on addressing either individual symptoms or so-called symptom clusters in cancer survivors, rather than authentic symptom clusters as defined. As per the conceptual framework of symptom clusters outlined by Dodd et al.^12^ the individual symptoms within a symptom cluster should be well confirmed through significant associations. The current systematic review followed this definition and provided the latest overview of the management of symptom clusters among BC survivors. Nonpharmacological interventions, such as acupressure, Tai Chi, physical exercise, and education programs, could be relatively safe and effective options for healthcare professionals to manage the BC survivors’ symptoms at a cluster level in clinical practice. However, due to the limited number of included studies and the methodological limitations of some included studies, further research based on our findings should be conducted to strengthen the evidence supporting the effects and cost-effectiveness of those nonpharmacological interventions and their applicability in a clinical setting. Large-scale, rigorously designed RCTs are warranted. Implementing a blinding design for outcome assessors and study participants is feasible. Moreover, it is advisable to consider the inclusion of biomarkers in addition to subjective outcomes (e.g., patient-reported scales) in future studies, as patients' expectations towards the study interventions could potentially impact the outcomes, especially in studies lacking adequate blinding and allocation concealment. Another reason comes from the significant relationships identified between symptom clusters and biomarkers related to inflammation (e.g., cytokines).^19^ Safety is also a vital consideration when implementing an intervention in clinical practice. While the findings of this review indicated that the nonpharmacological interventions have been well monitored for safety, none of the included RCTs provided any causality analysis between reported adverse events and targeted interventions. Future RCTs should include and report safety-related information by following the extension of the CONSORT statement on harms.^47^

### Limitations

This study has some limitations. Methodological issues such as ambiguous descriptions of allocation concealment and inadequate mask design in some studies are likely to induce a selection bias, which may lead to an overestimation of intervention effects, potentially affecting the reliability of the trial findings.^48^ Meanwhile, only English and Chinese literature were searched, leading to the possibility of language bias. Although grey literature and registered ongoing trials were included, this review was not able to ensure that all pertinent studies were tracked.

## Conclusions

This study identified that nonpharmacological interventions could be potentially considered as a beneficial way to manage the symptom clusters among BC survivors and improve their QoL. However, the findings of this review should be interpreted prudently due to the limited number of included studies and the methodological concerns identified in some studies. It is paramount to improve the quality of future RCTs by planning a rigorous methodology, adequate sample sizes, more extended follow-up periods, and causality analysis between the adverse event and the targeted interventions.

## CRediT author statement

**Meng-Yuan Li**: Conceptualization and methodology, literature search, data extraction and interpretation, manuscript drafting and revision. **Li-Qun Yao**: Conceptualization and methodology, literature search, data extraction and interpretation, manuscript drafting and revision. **Tao Wang**: Conceptualization and methodology, data interpretation, manuscript revision. **Xian-Liang Liu**: Conceptualization and methodology, manuscript revision. **Jing-Yu (Benjamin) Tan:** Conceptualization and methodology, manuscript revision. All authors had full access to all data in the study, and the corresponding author had final responsibility for the decision to submit for publication. The corresponding author attests that all listed authors meet authorship criteria and that no others meeting the criteria have been omitted.

## Declaration of competing interest

The authors declare no conflict of interest.

## Funding

This study was supported by the COVID-19 Supplementary Funding Pool (CSFP) Scheme at 10.13039/501100001803Charles Darwin University. The funders had no role in considering the study design or in the collection, analysis, interpretation of data, writing of the report, or decision to submit the article for publication.

## Ethics statement

Not required.

## Data availability statement

The authors confirm that the data supporting the findings of this study are available within the article and/or its supplementary materials.

## Declaration of Generative AI and AI-assisted technologies in the writing process

No AI tools/services were used during the preparation of this work.
