# Supplementary material for: Effects of nonpharmacological interventions on symptom clusters in breast cancer survivors: A systematic review of randomized controlled trials
Source: Asia Pac J Oncol Nurs. 2024 Jan 11;11(3):100380. doi: 10.1016/j.apjon.2024.100380 (PMC10909965; doi:10.1016/j.apjon.2024.100380)
Supplement: Multimedia component 2 [file mmc2.docx]

**Ten Electronic Databases Searching Strategy**

Mesh terms, keywords and free words

| Domain | Mesh terms | Key words and free words (used in English databases) | Key words and free words (used in Chinese databases) |
| --- | --- | --- | --- |
| Population | breast neoplasms | “breast neoplas*” or “breast tumo*” or “mammary neoplas*” or “breast cance*” or “breast carcinoma*” or “mammary cance*” or “mammary carcinoma*” or “breast malignant neoplas*” or “breast malignant tumo*” or “cancer of breast” | “乳腺肿瘤” or “乳腺癌” or “乳癌” or “乳腺瘤” |
| Outcome | syndrome | “syndrom*” or “symptom cluste*” or “symptom occurrence” or “concurrent symptom*” or “co-existing symptom*” or “multiple symptom*” | “症状集” or “症状群” or “症状簇” or “症候群” |
| Type of study design | “randomi?ed controlled trial as Topic” or “controlled clinical trial as a Topic” or **“controlled clinical trial [Publication Type]” or “randomised controlled trial[Publication Type]” or “randomized controlled trial[Publication Type]”** | “random*” or “control*” or “trial” | “随机” or “对照” or “控制” |

**1.PubMed**

| Search | Query |
| --- | --- |
| #1 | breast neoplasms[MeSH Terms] |
| #2 | **(((((((((breast neoplas*[Title/Abstract]) OR (breast tumo*[Title/Abstract])) OR (breast cance*[Title/Abstract])) OR (breast carcinoma*[Title/Abstract])) OR (mammary cance*[Title/Abstract])) OR (mammary neoplas*[Title/Abstract])) OR (mammary carcinoma*[Title/Abstract])) OR (breast malignant neoplas*[Title/Abstract])) OR (breast malignant tumo*[Title/Abstract])) OR (cancer of breast[Title/Abstract])** |
| #3 | **#1 OR #2** |
| #4 | **syndrome[MeSH Terms]** |
| #5 | **(((((syndrom*[Title/Abstract]) OR (symptom cluste*[Title/Abstract])) OR (symptom occurrence[Title/Abstract])) OR (concurrent symptom*[Title/Abstract])) OR (co-existing symptom*[Title/Abstract])) OR (multiple symptom*[Title/Abstract])** |
| #6 | **#4 OR #5** |
| #7 | **(((((randomised controlled trial[Publication Type]) OR (randomized controlled trial[Publication Type])) OR (clinical controlled trial[Publication Type])) OR (random*[Title/Abstract])) OR (control*[Title/Abstract])) OR (trial[Title/Abstract])** |
| #8 | #3 AND #6 AND #7 |
| #9 | **#3 AND #6 AND #7** Filters: **from 2001 - 2023** |

**2.Web of Science**

| Search | Search Query |
| --- | --- |
| #1 | **(((((((((TS=(breast neoplas*)) OR TS=(breast tumo*)) OR TS=(breast cance*)) OR TS=(breast carcinoma*)) OR TS=(mammary cance*)) OR TS=(mammary neoplas*)) OR TS=(mammary carcinoma*)) OR TS=(breast malignant neoplas*)) OR TS=(breast malignant tumo*)) OR TS=(cancer of breast)** |
| #2 | **(((((TS=(syndrom*)) OR TS=(symptom cluste*)) OR TS=(symptom occurrence)) OR TS=(concurrent symptom*)) OR TS=(co-existing symptom*)) OR TS=(multiple symptom*)** |
| #3 | **((((TI=(randomized controlled trial)) OR TI=(randomised controlled trial)) OR TI=(controlled clinical trial)) OR TI=(random*)) OR TI=(control*)** |
| #4 | #1 AND #2 AND #3 |
| #5 | Timespan: 2001-01-01 to 2023-08-017 |

**3.Cochrane**

| Search | Search Query |
| --- | --- |
| #1 | MeSH descriptor: [Breast Neoplasms] explode all trees |
| #2 | (breast neoplas*):ti,ab,kw OR (breast tumo*):ti,ab,kw OR (breast cance*):ti,ab,kw OR (breast carcinoma*):ti,ab,kw OR (mammary cance*):ti,ab,kw |
| #3 | (mammary neoplas*):ti,ab,kw OR (mammary carcinoma):ti,ab,kw OR (breast malignant neoplas*):ti,ab,kw OR (breast malignant tumo*):ti,ab,kw OR (cancer of breast):ti,ab,kw |
| #4 | #1 OR #2 OR #3 |
| #5 | (Syndrom*):ti,ab,kw OR (symptom cluste*):ti,ab,kw OR (symptom occurrence):ti,ab,kw OR (concurrent symptom*):ti,ab,kw OR (co-existing symptom*):ti,ab,kw |
| #6 | (multiple symptom*):ti,ab,kw |
| #7 | MeSH descriptor: [Syndrome] explode all trees |
| #8 | #5 OR #6 OR #7 |
| #9 | (controlled clinical trial):pt OR (randomi?ed control trial):pt OR (random*):pt OR (control*):ti,ab,kw |
| #10 | #4 AND #8 AND #9 |
| Trials matching #10 from2001-2023 | |

**4.** **CINAHL Ultimate**

| Search | Search Query |
| --- | --- |
| S1 | SU breast neoplasms OR SU breast neoplas* OR SU breast tumo* OR SU breast cance* OR SU breast carcinoma* OR SU mammary cance* OR SU mammary neoplas* OR SU mammary carcinoma* OR SU breast malignant neoplas* OR SU breast malignant tumo* OR SU cancer of breast |
| S2 | SU syndrom* OR SU symptom cluste* OR SU symptom occurrence OR SU concurrent symptom* OR SU multiple symptom* OR SU co-existing symptom* |
| S3 | PT randomi?ed controlled trials OR SU rct OR SU random* OR SU control* OR PT controlled clinical trial |
| S4 | S1 AND S2 AND S3 |
| S5 | S1 AND S2 AND S3  Limiters - Published Date: 20010101-20231231 Narrow by Language: - english |

**5. Ovid MEDLINE(R) ALL**

| Search | Search Query |
| --- | --- |
| #1 | Breast Neoplasms/ |
| #2 | (breast neoplas* or breast tumo* or breast cance* or breast carcinoma* or mammary cance* or mammary neoplas* or mammary carcinoma or breast malignant neoplas* or breast malignant tumo* or cancer of breast).mp. [mp=title, book title, abstract, original title, name of substance word, subject heading word, floating sub-heading word, keyword heading word, organism supplementary concept word, protocol supplementary concept word, rare disease supplementary concept word, unique identifier, synonyms, population supplementary concept word, anatomy supplementary concept word] |
| #3 | 1 or 2 |
| #4 | Syndrome/ |
| #5 | (syndrom* or symptom cluste* or symptom occurrence or concurrent symptom* or co-existing symptom* or multiple symptom*).mp. [mp=title, book title, abstract, original title, name of substance word, subject heading word, floating sub-heading word, keyword heading word, organism supplementary concept word, protocol supplementary concept word, rare disease supplementary concept word, unique identifier, synonyms, population supplementary concept word, anatomy supplementary concept word] |
| #6 | 4 or 5 |
| #7 | randomized controlled trial/ |
| #8 | (randomized controlled trial or controlled clinical trial or randomised controlled trial or random*).mp. [mp=title, book title, abstract, original title, name of substance word, subject heading word, floating sub-heading word, keyword heading word, organism supplementary concept word, protocol supplementary concept word, rare disease supplementary concept word, unique identifier, synonyms, population supplementary concept word, anatomy supplementary concept word] |
| #9 | 7 or 8 |
| #10 | 3 and 6 and 9 |
| #11 | limit 10 to yr="2001 -Current" |

**6. PsycINFO**

| Search | Search Query |
| --- | --- |
| S1 | SU breast neoplasms OR SU breast neoplas* OR SU breast tumo* OR SU breast cance* OR SU breast carcinoma* OR SU mammary cance* OR SU mammary neoplas* OR SU mammary carcinoma* OR SU breast malignant neoplas* OR SU breast malignant tumo* OR SU cancer of breast |
| S2 | SU syndrome OR SU symptom cluste* OR SU concurrent symptom* OR SU symptom occurrence OR SU co-existing symptom* OR SU multiple symptom* OR SU syndrom* |
| S3 | PT randomi?ed controlled trials OR PT controlled clinical trial OR SU random* OR SU rct OR SU control* OR SU trial |
| S4 | S1 AND S2 AND S3 |

**7. EMBase**

| Search | Search Query |
| --- | --- |
| #1 | 'breast tumor'/exp |
| #2 | 'breast tumo*':ti,ab,kw OR 'breast cance*':ti,ab,kw OR 'breast carcinoma*':ti,ab,kw OR 'mammary cance*':ti,ab,kw OR 'mammary neoplas*':ti,ab,kw OR 'mammary carcinoma':ti,ab,kw OR 'breast malignant neoplas*':ti,ab,kw OR 'breast malignant tumo*':ti,ab,kw OR 'human mammary neoplas*':ti,ab,kw OR 'breast neoplas*':ti,ab,kw |
| #3 | #1 OR #2 |
| #4 | syndrom*:ti,ab,kw OR 'symptom cluste*':ti,ab,kw OR 'symptom occurrence':ti,ab,kw OR 'concurrent symptom*':ti,ab,kw OR 'co-existing symptom*':ti,ab,kw OR 'multiple symptom*':ti,ab,kw |
| #5 | 'randomized controlled trial'/exp |
| #6 | 'randomized controlled trial':it OR 'randomised controlled trial':it OR 'controlled clinical trial':it OR random*:ti,ab,kw |
| #7 | #5 OR #6 |
| #8 | #3 AND #4 AND #7 |
| #9 | #3 AND #4 AND #7 AND [2001-2023]/py |

**8.CNKI**

( SU %= '乳腺癌' OR SU %= '乳腺肿瘤' OR SU %= '乳癌' OR SU %= '乳腺瘤' ) AND ( SU %= '症状群' OR SU %= '症状集' OR SU %= '症候群' OR SU %= '症状簇' ) AND ( SU %= '随机' OR SU %= '对照' OR SU %= '控制' ) 时间范围：2001-至今

**9.CBM**

| 4 | ((#3) AND ((#2) AND (#1)) AND 2001-[日期] |  |
| --- | --- | --- |
| 3 | "随机对照试验"[关键词:智能] OR "随机"[常用字段:智能] OR "对照"[常用字段:智能] OR "控制"[常用字段:智能] |  |
| 2 | "症状群"[常用字段:智能] OR "症状集"[常用字段:智能] OR "症候群"[常用字段:智能] OR "症状簇"[常用字段:智能] |  |
| 1 | "乳腺肿瘤"[加权:扩展] OR "乳癌"[常用字段:智能] OR "乳腺癌"[常用字段:智能] OR "乳腺瘤"[常用字段:智能] |  |

**10. WanFang Data**

[(((((((((((((主题=乳腺癌) OR 主题=乳腺肿瘤) OR 主题=乳癌) OR 主题=乳腺瘤))) AND (((主题=随机) OR 主题=对照))) AND (((((主题=症状群) OR 主题=症状簇) OR 主题=症状集) OR 主题=症候群))))))))](https://med.wanfangdata.com.cn/Paper/Search?q=(((((((((((((%E4%B8%BB%E9%A2%98=%E4%B9%B3%E8%85%BA%E7%99%8C)%20OR%20%E4%B8%BB%E9%A2%98=%E4%B9%B3%E8%85%BA%E8%82%BF%E7%98%A4)%20OR%20%E4%B8%BB%E9%A2%98=%E4%B9%B3%E7%99%8C)%20OR%20%E4%B8%BB%E9%A2%98=%E4%B9%B3%E8%85%BA%E7%98%A4)))%20AND%20(((%E4%B8%BB%E9%A2%98=%E9%9A%8F%E6%9C%BA)%20OR%20%E4%B8%BB%E9%A2%98=%E5%AF%B9%E7%85%A7)))%20AND%20(((((%E4%B8%BB%E9%A2%98=%E7%97%87%E7%8A%B6%E7%BE%A4)%20OR%20%E4%B8%BB%E9%A2%98=%E7%97%87%E7%8A%B6%E7%B0%87)%20OR%20%E4%B8%BB%E9%A2%98=%E7%97%87%E7%8A%B6%E9%9B%86)%20OR%20%E4%B8%BB%E9%A2%98=%E7%97%87%E5%80%99%E7%BE%A4))))))))&%E5%B9%B4%E4%BB%BD_fl=2001-2023&%E8%B5%84%E6%BA%90%E7%B1%BB%E5%9E%8B_fl=(%E4%B8%AD%E6%96%87%E6%9C%9F%E5%88%8A%20OR%20%E5%AD%A6%E4%BD%8D%E8%AE%BA%E6%96%87%20OR%20%E4%BC%9A%E8%AE%AE%E8%AE%BA%E6%96%87)&%E6%9C%9F%E5%88%8A%E6%A0%8F%E7%9B%AE_fl=(%E8%AE%BA%E8%91%97%20OR%20%E5%8E%9F%E8%91%97%20OR%20%E5%AE%9E%E9%AA%8C%E7%A0%94%E7%A9%B6%20OR%20%E4%B8%B4%E5%BA%8A%E7%A0%94%E7%A9%B6%20OR%20%E4%B8%B4%E5%BA%8A%E6%8A%A5%E5%91%8A%20OR%20%E7%8E%B0%E5%9C%BA)&SearchMode=Advanced)资源类型：(中文期刊 OR 学位论文 OR 会议论文) and Date:2001-*
